# The detection of *Mycoplasma sturni* and *Mycoplasma moatsii* from the choana of a barn swallow (*Hirundo rustica*): a case report

**DOI:** 10.1186/s12917-023-03589-1

**Published:** 2023-02-04

**Authors:** Theresa Sophie Klostermann, Michael Lierz

**Affiliations:** Clinic for Birds, Reptiles, Amphibians and Fish, Justus-Liebig-University Giessen, Frankfurter Str. 114, 35392 Giessen, Germany

**Keywords:** Commensals, Conjunctivitis, Free ranging birds, Mycoplasmas, Wild birds

## Abstract

**Background:**

Mycoplasmas are found in many different species. Until now 26 avian mycoplasma species have been described, but in the most free ranging bird species the prevalence and significance of *Mycoplasma* spp. is still unclear.

**Case presentation:**

In May 2021 a barn swallow (*Hirundo rustica*) was brought to a veterinary clinic after it hit a window. As part of the routine exam a choanal swab was taken for mycoplasma culture and for the detection of mycoplasmas using a *Mycoplasma*-genus-specific Polymerase chain reaction.

Six single colony subcultures were obtained by the cultivation. Obtained subcultures were investigated by sequencing the 16S rRNA and the 16S-23S rRNA intergenic transcribed spacer region sequence. The 16S rRNA gene sequence from one subculture had a homology of 99.03% and the 16S-23S rRNA intergenic transcribed spacer region sequence of 100% with the sequence of *Mycoplasma sturni.*

The 16S rRNA gene sequence from the other five subcultures shared a homology of 99.89% and the 16S-23S rRNA intergenic transcribed spacer region sequence of 99.81% with the sequence of *Mycoplasma moatsii.*

**Conclusions:**

According to the available literature this is the first report about the detection of *M. moatsii*, in the respiratory tract of a barn swallow. *M. moatsii* was previously only found in grivit monkeys (*Cercopithecus aethiops*), Norway rats (*Rattus norvegicus*) and a mute swan (*Cygnus olor*). The role of mycoplasmas in barn swallows is still unknown, especially as in the present case both mycoplasma species do not seem to cause clinical symptoms.

## Background

Mycoplasmas are found in many different species among them humans, mammals, birds, reptile, fish, insects and plants. Some mycoplasma species are highly host specific, where as others are not. Until now 26 avian mycoplasma species have been described. *Mycoplasma gallisepticum*, *Mycoplasma synoviae, Mycoplasma meleagridis*, and *Mycoplasma iowae* are relevant pathogens in domestic poultry and they are the cause of great economic losses [1]. In 1994 M*. gallisepticum* caused an epidemic conjunctivitis in the house finch (*Haemorhous mexicanus*) population in the USA with a high prevalence and a high mortality. The losses were up to 60% of the house finch population until the disease stabilized on a low prevalence level [2].

In the respiratory tract of healthy Passeriformes, *Mycoplasma* spp. have so far not been found. There is a hypothesis that species relying on vocal activity for reproduction, like Passeriformes, do not tolerate latent infections of the respiratory tract with mycoplasmas [3]. The infection with mycoplasmas would affect their voice and so reduce their reproductive success. Because of the reduced reproductive success of affected birds no evolutionary adaption to mycoplasmas has taken place [3]. Birds with a high prevalence of mycoplasmas like birds of prey, with prevalence’s about 91% to 94% [4], and storks, with a prevalence about 99% [5], don´t rely on their vocalization for reproduction [6, 7] and therefore can tolerate subclinical mycoplasma infection better. Corvids (*Corvidae*) are Passeriformes, but they use other strategies besides singing for mating [8]. They have an intermediate prevalence about 7% of mycoplasmas [9]. That supports the theory that the influence of singing for the reproduction success of birds and their mycoplasma prevalence is connected. This hypothesis might be the explanation for the differences in the mycoplasma prevalence in free ranging birds [3].

Barn swallows *(Hirundo rustica*) are Passeriformes and rely on vocal activity for reproduction, so in relation to this hypothesis the prevalence of mycoplasmas in the barn swallow population should be low. However, until now, only little is known about the prevalence and the significance of mycoplasmas in this species.

In different studies mycoplasmas have been demonstrated as part of the intestinal flora of juvenile and adult barn swallows [10–13]. On average 5.5% to 9.6% *Mycoplasmataceae* were detected in feces samples from barn swallows by molecular biological analysis, which shows a high amount of *Mycoplasmataceae* in the intestinal flora [12, 13].

*Mycoplasma sturni* was first isolated and described in 1996 from the eye of an adult European starling *(Sturnus vulgaris)* with bilateral conjunctivitis and moderate blepharitis [14]. Since then *M. sturni* was also isolated from eight northern mockingbirds *(Mimus polyglottos)* [15–17]*,* two blue jays *(Cyanocitta cristata)* [16, 17]*,* three scrub jays (*Aphelocoma californica)* [18], one House Finch, three European Starlings, twenty-one American Crows* (Corvus brachyrhynchos*), one American Robin (*Turdus migratorius*), one Carolina Wren (*Thryothorus ludovicianus*), seven Cliff Swallows (*Petrochelidon pyrrhonota*) and four Barn Swallows [17]. All these birds showed conjunctivitis. However co- infections with other pathogens could not be excluded in these studies [15–18].

Co-infections with other pathogens make it difficult to assess the pathogenic role of *M. sturni* and its role for the manifestation of the symptoms.

*M. sturni* was also isolated from four cliff swallow fledglings with clinical signs of ocular disease, characterized by conjunctivitis and mildly increased respiratory effort in a rehabilitation facility. One cliff swallow died and three were euthanized due to poor therapeutic response to the antibiotic therapy. A histopathologic examination demonstrated lymphoplasmacytic conjunctivitis, rhinitis and infraorbital sinusitis. Additionally *Cryptosporidium* spp. were found on the conjunctival, nasal and sinus epithelia [19]. It remains unclear, if the infection with *M. sturni* caused the conjunctivitis of the birds.

Moreover there are also findings of *M. sturni* in some birds without conjunctivitis.

In Scotland *M. sturni* was isolated from three blackbirds *(Turdus merula),* five rooks *(Corvus frugilegus)*, three carrion crows *(Corvus corone)*, two magpies (*Pica pica*) and five starlings. All the birds were immature and were suffering from a range of diseases, but showed no signs of conjunctivitis [20]. Additionally *M. sturni* was isolated from an American crow nestling with bilateral conjunctivitis in a Wildlife Rehabilitation Center and was also found in nine of eleven other juvenile asymptomatic American crows and in six asymptomatic American robins, which had contact to the bird with mycoplasma infection. Moreover *M. sturni* was isolated from a European starling without conjunctivitis, which was found dead in the aviary with the robins [21].

The first prevalence study detecting *M. sturni* in crows was performed in Germany using tracheal swabs from free-ranging 97 Corvids for *Mycoplasma* spp. culture and *Mycoplasma*-genus-specific Polymerase chain reaction (PCR). The samples were collected from 68 randomly selected adult individuals from hunting bags without clinical signs and 29 birds that had been admitted to a veterinary clinic. Of the hunted birds, five of 68 (7%) were positive and of the corvids admitted to the veterinary clinic nine of 29 (31%) were positive for *M. sturni*. Seven of these birds were juvenile Carrion Crows. Four of them were healthy. The others showed lameness, injuries or apathy. The other two positive birds were adult Carrion Crows showing fractures. The occurrence of *M. sturni* in the birds randomly selected by hunting was significantly lower than in the group, which was admitted to the veterinary clinic [9].

Moreover *M. sturni* failed to reproduce disease by experimental infection in a study using wild- caught House Finches. The conjunctival inoculation of a *M. sturni* isolate originating from a wildlife case with conjunctivitis resulted in transient infection in one of nine House finches, but no disease [22].

Therefore the clinical significance of *M. sturni* is still unclear, but it seems not to represent a primary pathogen.

*Mycoplasma moatsii* was first isolated and described in 1974 from healthy grivit monkeys (*Cercopithecus aethiops*), which were imported from Ethiopia to the United States. Five isolates were found in the reproductive tract and one isolate in the throat and the reproductive tract of these monkeys. It was than supposed that *M. moatsii* belongs to the microbiota of grivit monkeys [23]. In 1990 M*. moatsii* was isolated from the intestine of the progeny of wild Norway rats (*Rattus norvegicus*) (F2 generation), which were caught by traps in the north of Germany. It was identified by serological and biochemical tests. *M. moatsii* was not found in the intestine of SPF rats, which were investigated for mycoplasmas in the same study. Therefore it was supposed, that *M. moatsii* belongs to the indigenous microbial flora of wild Norway rats. It was also assumed that rats are the natural hosts of *M. moatsii* and that the grivit monkeys were infected with *M. moatsii* by rats [24]. Additionally *M. moatsii* was recently detected by PCR based on the 16S rRNA gene in a cloacal sample from a free- ranging mute swan without any clinical signs (*Cygnus olor*). It remains unclear, if this was a contaminant finding (intestinal passage) or an infestation as no culture was performed.

The sample was taken during a regular ringing action [25].

## Case presentation

### Sample information

In May 2021 an adult barn swallow was brought to our veterinary clinic after it hit a window. In the clinical examination the general condition and the nutrition status were good and the bird showed no signs of disease. A shoulder girdle fracture leading to the euthanasia of the bird was detected. As part of the routine exam a choanal swab was taken for the detection of mycoplasmas with PCR and for mycoplasma culture.

### Mycoplasma culture

The choanal swab was immersed in 2 ml AL10 medium (Avian Mycoplasma Liquid Medium; Mycoplasma Experience Ltd, Bletchingley, United Kingdom) before it was removed and stored at − 80 °C until further investigation by PCR. For the cultivation the liquid medium, in which the swab was immersed, was diluted two times. 200 µl of the original suspension was given in a tube with 2 ml AL10. From that first dilution 200 µl was given in another tube with 2 ml AL10. An aliquot of 25 μL of each dilution was transferred onto AS1 agar (Avian Mycoplasma Agar & Supplement; Mycoplasma Experience Ltd). Liquid and solid media were incubated at 37 °C with 5% CO_2_ in a humidified environment for up to 15 days (d). Sub culturing of liquid broth on agar plates was performed after 5 d and 10 d. The broth was examined daily for color change and agar plates for colony growth. After 5 days mycoplasma typical colonies were detected and single colony subcultures were performed to ensure pure cultures. The selection criteria were a typical egg fried appearance and that the colonies lay separately from other colonies. These procedures were repeated twice resulting in six single colony subcultures.

### PCR and sequencing

The choanal swab and the six single colony subcultures were used for molecular biological analysis.

For DNA extraction the choanal swab was soaked and rubbed in 350 μL of PBS (phosphate-buffered saline). 100 μL of the liquid was used for DNA extraction using the DNeasy blood & tissue kit (Qiagen, Hilden, Germany) according to the manufacturer's instructions. For DNA extraction from the single-colony subcultures, the fluid medium (1 mL) was centrifuged at 4,000 × G for 45 min. The remaining pellet was incubated with 180 μL of lysis buffer (ATL buffer, Qiagen) and 20 μL of proteinase K (Qiagen) for 2 h at 56 °C. 100 μL of the liquid was used for DNA extraction using the DNeasy blood & tissue kit (Qiagen) according to the manufacturer's instructions. The concentration of the DNA of each sample was measured via spectrophotometer (Nanodrop 2000, Thermo Scientific, Wilmington, Delaware, USA) and, if necessary, diluted to 10 ng/μL. The extracted DNA of the swab and the single-colony subcultures were screened via *Mycoplasma*-genus-specific PCR targeting the 16S ribosomal RNA gene sequence as described by van Kuppeveld et al. [26] modified by Lierz et al. [27]. Also an additional PCR, targeting the 16S-23S rRNA intergenic transcribed spacer region (ISR) sequence as described by Ramírez et al. [28], was performed from the DNA of any single-colony subculture. 5 μl of each amplified product was mixed with 3 μl Loading dye (Thermo Fisher Scientific; Waltham, USA) and was separated by electrophoresis on a 1% agarose gel stained with GelRed™. As DNA Marker GeneRuler 100 bp DNA Ladder (Thermo Fisher Scientific; Waltham, USA) was used. The amplicons were visualized with ultraviolet light.

The 16S ribosomal RNA gene sequence from the single-colony subcultures and the 16S-23S rRNA intergenic transcribed spacer region sequence were purified with the GeneJET PCR Purification KIT (Thermo Fisher Scientific) and sequenced by LGC Genomics (Berlin, Germany). The sequences were viewed with BioEdit and analyzed with nucleotide BLAST (Basic Local Alignment Search Tool) by NCBI.

### Results

**Fig. 1:**
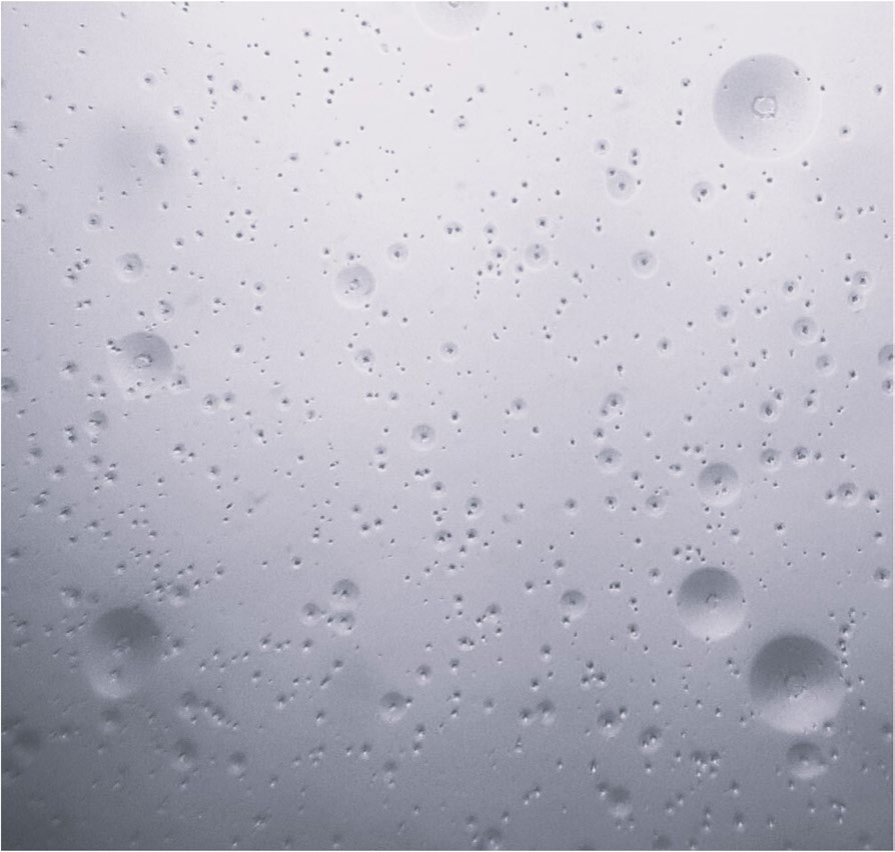
Colonies from the cultivation of the choanal swab with typical fried-egg appearance

Six isolates of mycoplasmas as single-colony subcultures in the third passage were obtained by cultivation. Five of these isolates had a similar morphology and one isolate had a little bit larger colonies than the others. All colonies showed a typical fried-egg appearance (Fig. 1). Each isolate was stored at − 80 °C.

The isolates of 5 subcultures grew rather slow. The isolate with larger colonies grew faster. The liquid medium showed a color change from red to yellow after two or three days due to a glucose metabolism of the isolates.

**Fig. 2:**
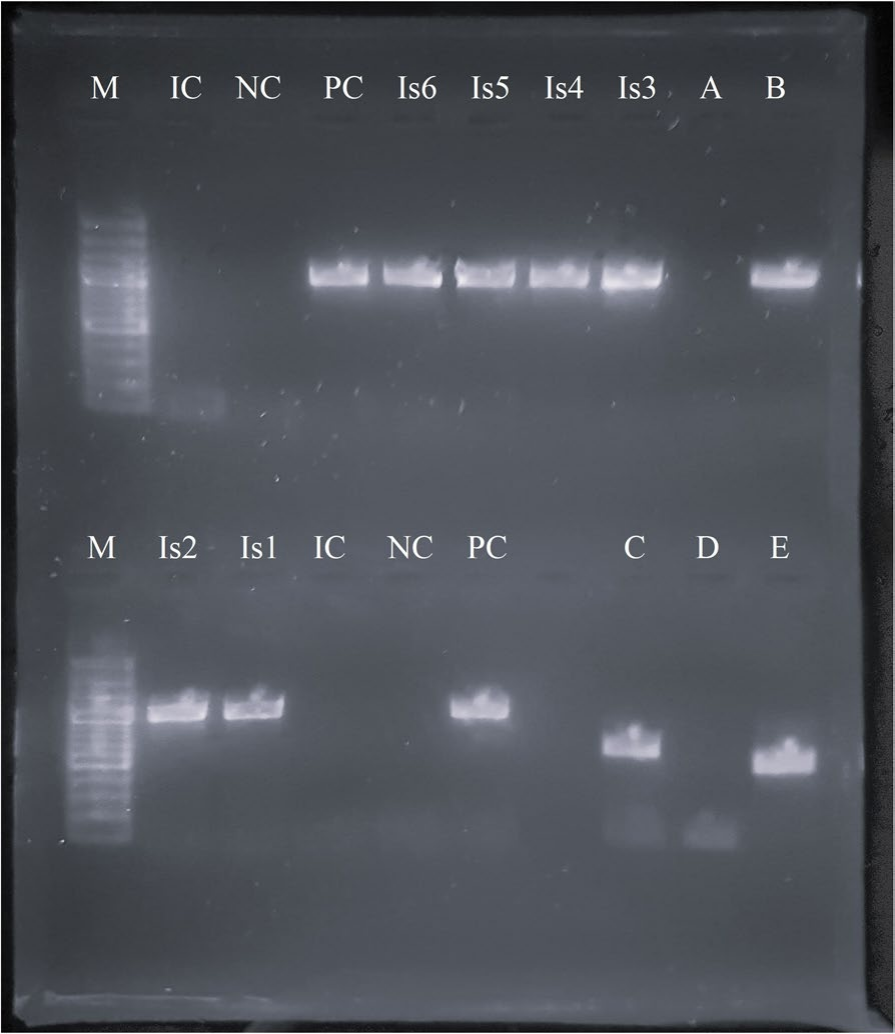
Picture of the gel from the electrophorese of the 16S rRNA PCR products from the isolates. The 16S rRNA PCR products had a size of approximately 1000 bp, of which were sequenced products with 923 bp (Is3) or 936 bp (Is1, Is2, Is4, Is5, Is6) respectively. Isolate 3 showed larger colonies in cultivation and turned out as *M. sturni*. Lanes A to E are not related to the present study.(M = DNA Marker, Is = Isolate, PC = positive control, NC = negative control, IC = internal control)

**Fig. 3:**
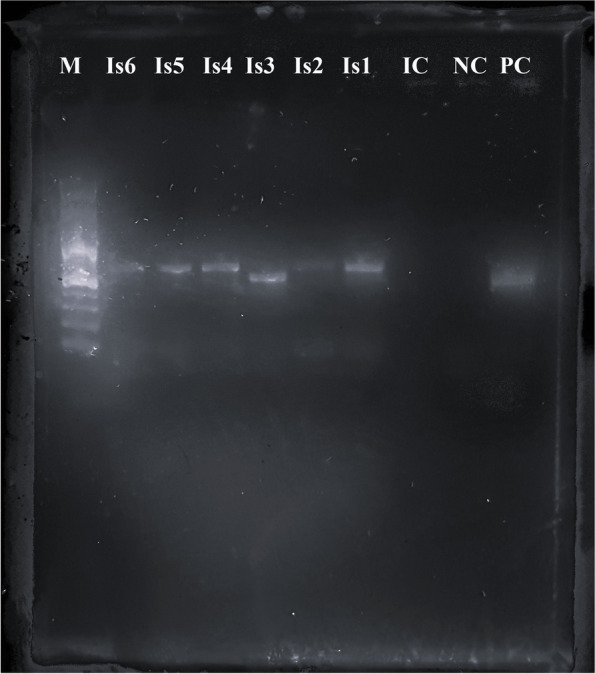
Picture of the gel from the electrophorese of the 16S-23S rRNA intergenic transcribed spacer region PCR products from the isolates. The PCR products had a size of approximately 500 bp (Is3) or 600 bp (Is1, Is2, Is4, Is5, Is6) respectively, of which were sequenced products with 443 bp (Is3) or 517 bp (Is1, Is2, Is4, Is5, Is6) respectively. Isolate 3 showed larger colonies in cultivation and turned out as *M. sturni*.(M = DNA Marker, Is = Isolate, PC = positive control, NC = negative control, IC = internal control)

The *Mycoplasma*-genus-specific PCR from the swab and the six isolates (Fig. 2) detected *Mycoplasma* spp.*.* Also the PCR targeting the 16S-23S rRNA ISR sequence (Fig. 3) from the six isolates demonstrated a *Mycoplasma* spp. typical PCR product.

The sequence of the 16S rRNA gene (GenBank Accession No: ON529526) of the isolate, which showed larger colonies in the cultivation, had a query coverage of 100% and a similarity of 99.03% with the 16S ribosomal RNA of *M. sturni* (GenBank Accession No: NR_025968.1). The next similar mycoplasma species were *Mycoplasma columboralis* (GenBank Accession No: NR_025179.1) with a query coverage of 99% and a similarity of 96.41% and *Mycoplasma citelli* (GenBank Accession No: NR_025178.1) with a query coverage of 100% and a similarity of 95.34%. The 16S-23S rRNA ISR sequence (GenBank Accession No: ON540700) had a query coverage of 100% and a similarity of 100% with the 16S-23S rRNA ISR sequence of *M. sturni* (GenBank Accession No: AY766090.1), so the sequences were identical. Due to the high homology of the sequences this isolate was diagnosed as *M. sturni.*

The 16S rRNA gene sequences and the 16S-23S rRNA intergenic transcribed spacer region sequences from the five isolates with the same morphology appeared very similar and were adjusted to the same length with 936 bp (basepair) or 517 bp respectively. The alignment of the 16S rRNA gene sequences showed a similarity of 100% to each other and also the 16S-23S rRNA intergenic transcribed spacer region sequences were 100% identical to each other. The sequence of the 16S ribosomal RNA gene (GenBank Accession No: ON527780) from the 5 isolates had a query coverage of 100% and a similarity of 99.89% with the 16S ribosomal RNA of *M. moatsii* (GenBank Accession No: NR_025186.1). The next similar mycoplasma species were *Mycoplasma sualvi* (GenBank Accession No: NR_041846.1) with a query coverage 100% and a similarity of 96.58% and *Mycoplasma mobile* (GenBank Accession No: NR_074620.2) with a query coverage of 99% and a similarity of 90.79%. Additionally the sequence of the 16S ribosomal RNA gene from *M. moatsii* detected in a cloacal swab from a Mute Swan (GenBank accession no. MT374252) and the 16S rRNA of the barn swallow were aligned with a similarity of 98.12%.

The 16S-23S rRNA ISR sequence (GenBank Accession No: ON540701) had a query coverage of 100% and a similarity of 99.81% with the 16S-23S rRNA ISR sequence of *M. moatsii* (GenBank Accession No: DQ847422.1). The next similar mycoplasma species were *M. sualvi* (GenBank Accession No: AY766089.1) with a query coverage of 100% and a similarity of 80.30% and *Mycoplasma langogenitalium* (GenBank Accession No: AY785379.1) with a query coverage of 24% a similarity of 95.31%. Due to the high homology of the sequences the five isolates were regarded as *M. moatsii*.

## Discussion and conclusions

We isolated *M. sturni* and *M. moatsii* from the choana of a barn swallow. The 16S rRNA gene sequences of all isolates showed a very high similarity to the 16S rRNA gene sequence of *M. sturni or M. moatsii* respectively. The 16S rRNA gene sequences are considered to be a very good tool for taxonomic classification and are one of the obligatory requirements for the description of new Mollicutes species [29, 30]. Additionally, the 16S-23S rRNA intergenic transcribed spacer region sequences show a very high similarity to the 16S-23S rRNA ISR sequence of *M. sturni or M. moatsii*. The 16S-23S rRNA ISR sequence is a good value for determining species since there is a high inter-species variation between avian mycoplasma species, and additionally there is low intra-species variation [28].

It is proposed that strains with a sequence similarity of the 16S rRNA gene above 97% belong most likely to the same species. Furthermore an arbitrary similarity value around 95–98% is proposed for the 16S-23S rRNA ISR sequence [30].

The similarity of both the 16S rRNA gene sequence and the 16S-23S rRNA intergenic transcribed spacer region sequence compared with the sequences of *M. sturni* or *M. moatsi* respectively are above these values for all isolates.

So although we didn´t use serological or biochemical tests for the identification of the isolates, as the specific antisera are not available, we confirm the identity of one subculture as *M. sturni* and the other five subcultures as *M. moatsii*, because of the very high similarity of the gene sequences to *M. sturni* or *M. moatsii* respectively.

In this case the barn swallow showed neither conjunctivitis nor respiratory symptoms nor other clinical signs. Although the pathogenic role of *M. sturni* for Passeriformes remains unclear, this case indicates that *M. sturni* or at least some *M. sturni* strains could be commensals or contaminants in barn swallows and other Passeriformes.

According to the available literature there are no previous reports about *M. moatsii* in barn swallows, so we assume that this is the first report about the detection of *M. moatsii* from the respiratory tract of a barn swallow.

In our study *M. moatsii* was isolated from the choana showing a clear infection of the bird. Therefore the present results show that *M. moatsii* occurs in birds. It is likely that barn swallows are carrier for *M. moatsii*, especially as the patient of this study has not shown any clinical signs.

Barn swallows are afro-palearctic migratory birds, which have breeding habitants in Germany and they cross Ethiopia on their way to their winter habitats [31]. Therefore a transmission of *M. moatsii* between grivit monkeys and barn swallows in Ethiopia and also between barn swallows and rats in Germany might be possible. It is known that flies can carry mycoplasmas [32] and those might play an additional role in the transmission. Mute Swans have a great distribution in Europe, so there exists also a potential contact between mute swans and barn swallows. There are reports about high prevalences of mycoplasmas in wild waterfowl without clinical signs. In a study with free ranging Great White Pelicans (*Pelecanus onocrotalus*) without clinical signs from the Western Cape, South Africa, 98% were tested positive for mycoplasmas, but the mycoplasma species were not specified [33]. Sawicka-Durkalec et al. found a high occurrence of *Mycoplasma* spp. in free ranging common gulls (*Larus canus*) (*N* = 111; 97.3%), European herring gulls (*Larus argentatus*) (*N* = 16; 93.8%), black-headed gulls (*Chroicocephalus ridibundus*) (*N* = 84; 79.8%), great cormorants (*Phalacrocorax carbo*) (*N* = 37; 51.4%), and mallards (*Anas platyrhynchos*) (*N* = 326; 36.8%). All birds showed no clinical signs of disease [25]. Therefore it is a possibility that *M. moatsii* is a commensal or contaminant in mute swans and barn swallows are the carrier. We found *M. sturni* and *M. moatsii* in the choana of a barn swallow without any clinical signs and in a good condition, which likely indicates that it was a latent infection.

An impairing eye sight of the barn swallow in this study cannot completely be excluded, despite it was not recognized in the clinical examination. *M. sturni* and *M. moatsii* could therefore potentially be opportunistic pathogen in barn swallows, which at this point seems very unlikely. However, until the wild population of barn swallows has been studied, the pathogenicity of *M. sturni* and *M. moatsii* and the role of barn swallows as carrier for *Mycoplasma* spp. remains unclear. Generally, it is still not much known about the prevalence and significance of *Mycoplasma* spp. in free ranging birds, but the present case shows that such investigations are necessary, especially prevalence studies, to allow a better interpretation of single findings of mycoplasmas in birds.

## Data Availability

The datasets generated and/or analysed during the current study are available in the Genbank database by NCBI. GenBank Accession No: ON529526; ON527780; ON540700; ON540701.

## Abbreviations

Bp: Basepair
BLAST: Basic Local Alignment Search Tool
d: Days
ISR: Intergenic transcribed spacer region
PCR: Polymerase chain reaction
PBS: Phosphate-buffered saline

## References

[1] Ferguson-Noel N, Armour NK, Noormohammadi AH, El-Gazzar M, Bradbury, JM. Mycoplasmosis. In: Diseases of Poultry. Swayne DE, Boulianne M, Logue CM, McDougald LR, Nair V, Suarez DL, Wit S, Grimes T, Johnson D, Kromm M, Prajitno TV, Rubinoff I, Zavala G editors. Wiley-Blackwell by John Wiley & Sons Inc., Ames, Iowa, 2020; pp. 907–965. 10.1002/9781119371199.ch21.

[2] Hochachka WM, Dobson AP, Hawley DM, Dhondt AA (2021). Host population dynamics in the face of an evolving pathogen. J Anim Ecol.

[3] Fischer L, Möller Palau-Ribes F, Kipper S, Weiss M, Landgraf C, Lierz M (2021). Absence of Mycoplasma spp. in nightingales (Luscinia megarhynchos) and blue (Cyanistes caeruleus) and great tits (Parus major) in Germany and its potential implication for evolutionary studies in birds. Eur J Wildl Res.

[4] Lierz M, Hagen N, Hernadez-Divers SJ, Hafez HM (2008). Occurrence of mycoplasmas in free-ranging birds of prey in Germany. J Wildl Dis.

[5] Möller Palau-Ribes F, Enderlein D, Hagen N, Herbst W, Hafez HM, Lierz M (2016). Description and prevalence of Mycoplasma ciconiae sp. nov. isolated from white stork nestlings (Ciconia ciconia). Int J Syst Evol Microbiol.

[6] Palokangas P, Alatalo RV, Korpimäki E (1992). Female choice in the kestrel under different availability of mating options. Anim Behav.

[7] Bocheński M, Jerzak L, Tryjanowski P, Sparks TH, Jerzak L (2006). Behaviour of the white stork Ciconia ciconia: a review. The White Stork in Poland: Studies in Biology, Ecology and Conservation.

[8] Clayton NS, Emery NJ (2007). The social life of corvids. Curr Biol.

[9] Ziegler L, Palau-Ribes FM, Schmidt L, Lierz M (2017). Occurrence and relevance of Mycoplasma sturni in free-ranging corvids in Germany. J Wildl Dis.

[10] Kenzaka T, Hozzein WN (2018). Dissemination of Intestinal Microbiota by Migratory Birds across Geographical Borders. Metagenomics-Basics, Methods and Applications.

[11] Ambrosini R, Corti M, Franzetti A, Caprioli M, Rubolini D, Motta VM (2019). Cloacal microbiomes and ecology of individual barn swallows. FEMS Microbiol Ecol.

[12] Kenzaka T, Tani K, Kumavath R (2017). Public Health Implications of Intestinal Microbiota in Migratory Birds. Metagenomics for Gut Microbes.

[13] Kreisinger J, Kropáčková L, Petrželková A, Adámková M, Tomášek O, Martin JF, Michálková R, Albrecht T. Temporal Stability and the Effect of Transgenerational Transfer on Fecal Microbiota Structure in a Long Distance Migratory Bird. Front Microbiol. 2017;8(50). 10.3389/fmicb.2017.00050. 10.3389/fmicb.2017.00050 PMC5292904 28220109

[14] Forsyth MH, Tully JG, Gorton TS, Hinckley L, Frasca S, Van Kruiningen HJ, Geary SJ (1996). Mycoplasma sturni sp. Nov., from the Conjunctiva of a European Starling (Sturnus vulgaris). J Syst Evol Microbiol.

[15] Frasca S, Hinckley L, Forsyth MH, Gorton TS, Geary SJ, Kruiningen HJV (1997). Mycoplasmal Conjunctivitis in a European Starling. J Wildl Dis.

[16] Ley DH, Geary SJ, Berkhoff JE, McLaren JM, Levisohn S (1998). Mycoplasma sturni from Blue Jays and Northern Mockingbirds with Conjunctivitis in Florida. J Wildl Dis.

[17] Ley DH, Hawley DM, Geary SJ, Dhondt AA (2016). House Finch (Haemorhous mexicanus) Conjunctivitis, and Mycoplasma spp. Isolated from North American Wild Birds, 1994–2015. J Wildl Dis.

[18] Rogers KH, Ley DH, Woods LW (2019). Mycoplasmosis of House Finches (Haemorhous mexicanus) and California Scrub-Jays (Aphelocoma californica) in a Wildlife Rehabilitation Facility with Probable Nosocomial Transmission. J Wildl Dis.

[19] Ley DH, Moresco A, Frasca S (2012). Conjunctivitis, rhinitis, and sinusitis in cliff swallows (Petrochelidon pyrrhonota) found in association with Mycoplasma sturni infection and cryptosporidiosis. Avian Pathol.

[20] Pennycott TW, Dare CM, Yavari CA, Bradbury JM (2005). Mycoplasma sturni and Mycoplasma gallisepticum in wild birds in Scotland. Vet Rec.

[21] Wellehan JF, Calsamiglia M, Ley DH, Zens MS, Amonsin A, Kapur V (2001). Mycoplasmosis in captive crows and robins from Minnesota. J Wildl Dis.

[22] Ley DH, Anderson N, Dhondt KV, Dhondt AA (2010). Mycoplasma sturni from a California House Finch with Conjunctivitis Did Not Cause Disease in Experimentally Infected House Finches. J Wildl Dis.

[23] Madden DL, Moats KE, London WT, Matthew EB, Sever JL (1974). Mycoplasma moatsii, a New Species Isolated from Recently Imported Grivit Monkeys (Cercopithecus aethiops). Int J Syst Evol Microbiol.

[24] Giebel J, Binder A, Kirchhoff H (1990). Isolation of Mycoplasma moatsii from the intestine of wild Norway rats (Rattus norvegicus). Vet Microbiol.

[25] Sawicka-Durkalec A, Kursa O, Bednarz Ł, Tomczyk G (2021). Occurrence of Mycoplasma spp. in wild birds: phylogenetic analysis and potential factors affecting distribution. Sci Rep.

[26] Kuppeveld FJv, Logt JTvd, Angulo AF, Zoest MJv, Quint WG, Niesters HG, Galama JM, Melchers WJ (1992). Genus- and species-specific identification of mycoplasmas by 16S rRNA amplification. Appl Environ Microbiol.

[27] Lierz M, Hagen N, Harcourt-Brown N, Hernandez-Divers SJ, Lüschow D, Hafez HM (2007). Prevalence of mycoplasmas in eggs from birds of prey using culture and a genus-specific mycoplasma polymerase chain reaction. Avian Pathol.

[28] Ramírez AS, Naylor CJ, Pitcher DG, Bradbury JM (2008). High inter-species and low intra-species variation in 16S–23S rDNA spacer sequences of pathogenic avian mycoplasmas offers potential use as a diagnostic tool. Vet Microbiol.

[29] Brown DR, Whitcomb RF, Bradbury JM (2007). Revised minimal standards for description of new species of the class Mollicutes (division Tenericutes). Int J Syst Evol Microbiol.

[30] Volokhov DV, Simonyan V, Davidson MK, Chizhikov VE (2012). Mol Phylogenet Evol.

[31] Briedis M, Kurlavičius P, Mackevičienė R, Vaišvilienė R, Hahn S (2018). Loop migration, induced by seasonally different flyway use, in Northern European Barn Swallows. J Ornithol.

[32] Gioia G, Freeman J, Sipka A, Santisteban C, Wieland M, Gallardo VA, Monistero V, Scott JG, Moroni P (2022). Pathogens associated with houseflies from different areas within a New York State dairy. JDS Commun.

[33] Assunção P, de Ponte Machado M, De la Fe C, Ramírez AS, Rosales RS, Antunes NT, Poveda C, Poveda JB (2007). Prevalence of Pathogens in Great White Pelicans (Pelecanus onocrotalus) from the Western Cape, South Africa. J Appl Anim Res.

